# Postmenopausal Vaginal Microbiome and Microbiota

**DOI:** 10.3389/frph.2021.780931

**Published:** 2022-01-14

**Authors:** Nayara Santos de Oliveira, Ana Beatriz Feijão de Lima, Juliana Carvalho Regino de Brito, Ayane Cristine Alves Sarmento, Ana Katherine Silveira Gonçalves, José Eleutério

**Affiliations:** ^1^Postgraduate Program in Pathology, Federal University of Ceará (UFC), Fortaleza, Brazil; ^2^Eleutério Laboratory, Fortaleza, Brazil; ^3^Health Sciences Postgraduate Program, Federal University of Rio Grande Do Norte (UFRN), Natal, Brazil; ^4^Department of Obstetrics and Gynecology, Federal University of Rio Grande Do Norte (UFRN), Natal, Brazil; ^5^Department of Obstetrics and Gynecology, Federal University of Ceará, Fortaleza, Brazil

**Keywords:** menopause, vulvovaginal atrophy, microbiome, microbiota, lactobacilli

## Abstract

The ovulatory cycle has a significant influence on the microbial composition, according to the action of estrogen and progesterone on the stratified squamous epithelium, due to an increase in epithelial thickness, glycogen deposition, and influence on local immunology. The 16S rRNA gene amplification and pyrosequencing study demonstrated that healthy women have community state types (CST), classified as; type “*L*,” with a predominance of *Lactobacillus crispatus*, type II, with a predominance of *Lactobacillus gasseri*, type III, where *Lactobacillus iners* predominates, and type V with a predominance of *Lactobacillus jensenii*. Type IV does not identify lactobacilli but a heterogeneous population of bacteria. There seems to be a relationship between increased vaginal bacterial diversity and poverty of lactobacilli with the complaining of vaginal dryness. With menopause, there appears to be a reduction in lactobacilli associated with higher serum levels of follicle-stimulating hormone (FSH) and lower estrogen levels. The evaluation of Gram-stained vaginal smears in postmenopause women must take into account the clinical-laboratory correlation. We should observe two meanly possibilities, atrophy with few bacterial morphotypes, without inflammatory, infiltrate (atrophy without inflammation), and atrophy with evident inflammatory infiltrate (atrophy with inflammation or atrophic vaginitis). The relationship between the microbiome and postmenopausal vulvovaginal symptoms seems to be related to the bacterial vaginal population. However, more robust studies are needed to confirm this impression.

## Introduction

Human beings are considered holobionts, a set of host organisms with stable and transient symbiotic microbiota. Therefore, the interaction between the host and its microbiome in a balanced way is a significant health maintenance factor (1). It is essential to define what a microbiome is and what a microbiota is. The latter terms are not synonymous. A microbiome is the collection of all microorganism genomes in a given ecosystem, while a microbiota consists of the microorganisms in that ecosystem themselves (2).

The vaginal microbiome and microbiota have been extensively studied, and their constitution is dynamic and related to age, ethnicity, use of medications, sexual habits. Vaginal dysbiosis is an imbalance in this microenvironment associated with complications such as pelvic inflammatory disease, increased risk of infection of the human immunodeficiency virus (HIV), human papillomavirus (HPV), in addition to poor repercussions on pregnancy. During the reproductive period, the vaginal microbiota can be dominated by species of lactobacilli, and eventually, by a polymicrobial flora without lactobacilli. The ovulatory cycle has a significant influence on the microbial composition, according to the action of estrogen and progesterone on the stratified squamous epithelium, due to an increase in epithelial thickness, glycogen deposition, and influence on local immunology (3).

## Menopause

Menopause is the arrest of menstrual cycles, associated with ovarian failure with a drop in estrogen secretion and an absence of progesterone secretion. With increased life expectancy, the phase after menopause can correspond to a third of a woman's life. The lack of estrogenic action restricts the squamous epithelium to the basal and parabasal layers, poor glycogen. There is a significant association between bacterial composition and vaginal atrophy (4).

## Vaginal Microbiome

The 16S rRNA gene amplification and pyrosequencing study demonstrated that there are community state types (CST). Such communities were classified as; type I, with a predominance of *Lactobacillus crispatus*, type II, with a predominance of *Lactobacillus gasseri*, type III, where *Lactobacillus iners* predominates, and type V with a predominance of *Lactobacillus jensenii*. Type IV does not identify lactobacilli but a heterogeneous population of bacteria. IV-A is composed of a small proportion of lactobacilli and a proportion of anaerobic bacteria (*Anaerococcus, Peptoniphilus*, and *Prevotella*). Moreover, type V-B, with a large proportion of *Atopobium*, in addition *to Prevotella, Parvimonas, Sneathia, Gardnerella, Mobiluncus*, or *Peptoniphilus* (5, 6).

## Vaginal Microbiome and Menopause

Women who have a CST III vaginal community in the perimenopausal period seem to have a greater propensity for a transition to CST IV-A in the postmenopausal period, which, in turn, would be more associated with atrophic vaginitis (4). Hummelen et al. (7) consider that there seems to be a relationship between increased vaginal bacterial diversity and poverty of lactobacilli with the complaining of vaginal dryness. However, Mitchell et al. (8) did not observe a correlation between genitourinary symptoms and characteristics of the vaginal microbiota. More recently, Wang et al. (9), studying Korean women, observed a significant decrease in the bacterial species and that the lactic-acid concentration also decreases.

## Vaginal Microbiota and Menopause

**Figure 1:**
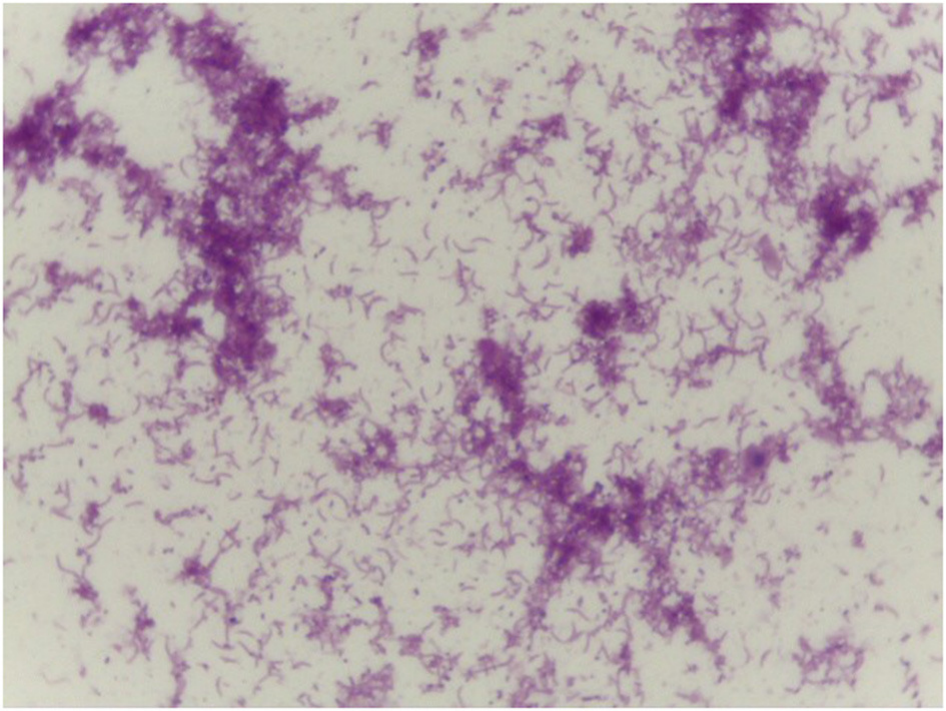
Curved Gram-negative bacillus in the vaginal smear, similar to *Mobiluncus* sp (Gram 1000x).

The study of the microbiome by pyrosequencing is still very far from most services in the world. In everyday life, more traditional methods are still used to study the vaginal microbiota, such as Gram bacterioscopy. In menacme and perimenopause, medium and short Gram-positive bacilli are more frequent, and in the event of dysbiosis, a polymicrobial population predominates. With menopause, there appears to be a reduction in lactobacilli associated with higher serum levels of follicle-stimulating hormone (FSH) and lower estrogen levels (9). Intriguingly, more recently, researchers observed that in cases of postmenopausal women complaining of vaginal dryness and irritation, there is a significant frequency in the vagina of BVAB1, which morphologically presented as a curved Gram-negative bacillus, similar to *Mobiluncus* sp. (8) (Figure 1).

**Figure 2:**
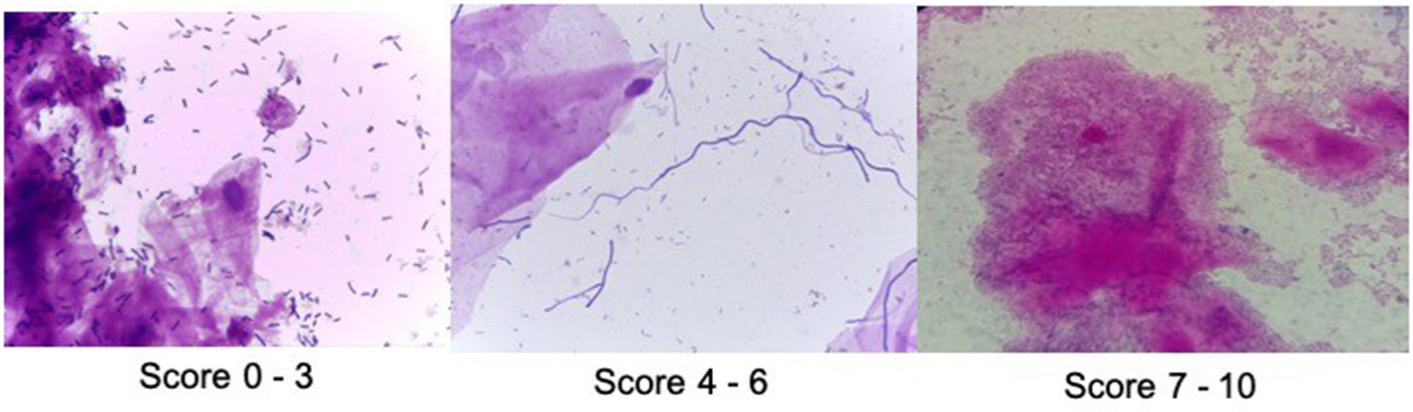
Examples of vaginal microbiota–Nugent score (Gram 1000x).

**Figure 3:**
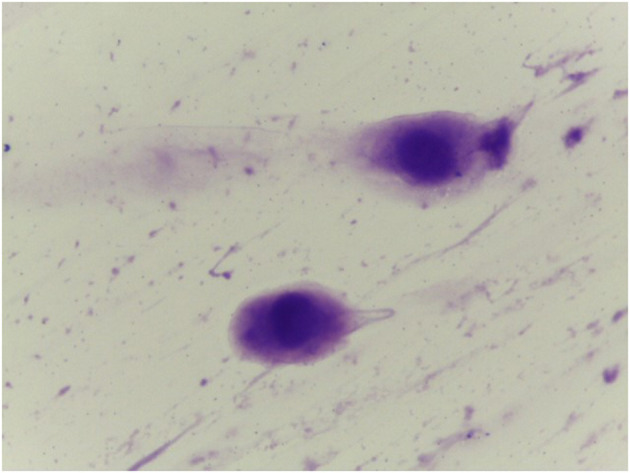
Vaginal smear in case of atrophy without inflammation (Gram 1000x).

**Figure 4:**
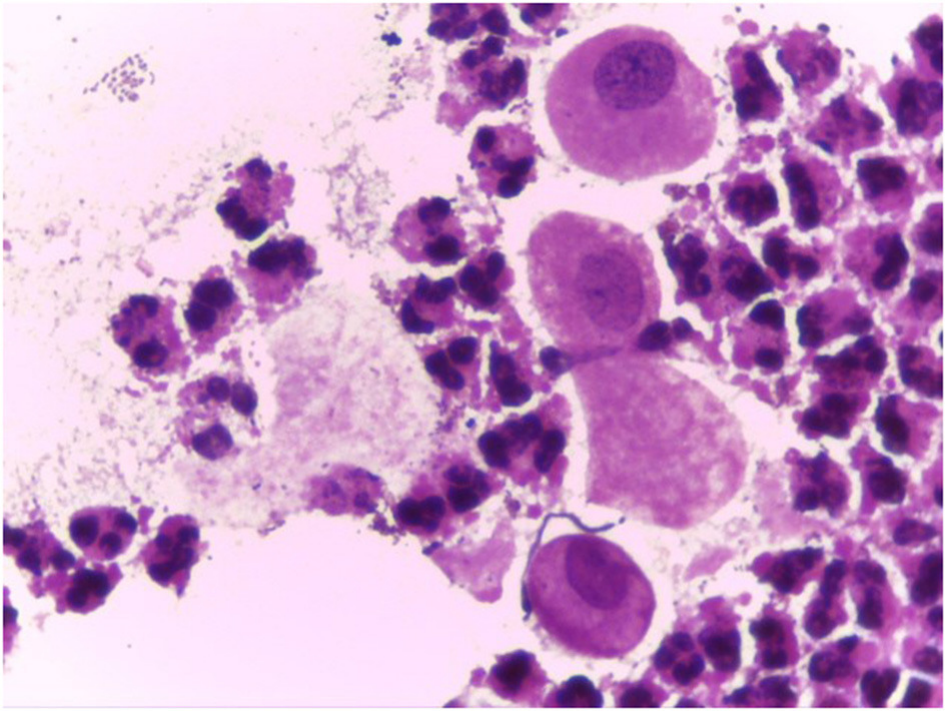
Vaginal smear in case of atrophy with inflammation (atrophic vaginitis) (Gram 100X).

The study of vaginal microbiota is often performed using the Nugent score (10) considered the gold standard for the diagnosis of bacterial vaginosis. This method evaluates three bacterial morphotypes. Gram-positive medium bacilli, short bacilli/Gram-variable *coccobacillus*, and Gram-negative curved bacilli. The morphotypes are quantified, and values are applied and added together to form the score. This evaluation, however, does not take into account the heterogeneity of the bacterial population, the formation of biofilm morphotypes, the inflammatory infiltrate, or the presence of parabasal cells. In menacme, scores can range from 0 to 10, with 0 to 3 considered normal, 4 to 6 considered intermediate, and 7 to 10 diagnosis of bacterial vaginosis (Figure 2). In post-menopause with estrogen deficit, in normal conditions, or atrophy cases with inflammation, the score gives little information. In both situations, the score tends to be between 4 and 6, that is, intermediate. The evaluation of Gram-stained vaginal smears may help, taking into account the clinical-laboratory correlation. We should observe two meanly possibilities, atrophy with few bacterial morphotypes, without inflammatory, infiltrate (atrophy without inflammation) (Figure 3) and atrophy, which even with few morphotypes, presents an evident inflammatory infiltrate (atrophy with inflammation or atrophic vaginitis) (Figure 4).

## Conclusion

The postmenopausal estrogen deficit influences the vaginal microbiome, reducing the number of lactobacilli. The reduction in the number of lactobacilli in the vagina follows the decrease in serum estrogen levels. The relationship between the microbiome and postmenopausal vulvovaginal symptoms seems to be related to the bacterial vaginal population. However, more robust studies are needed to confirm this impression. However, the microbiota analysis with Nugent score to assess bacterial vaginal community in the atrophic vaginitis is not very helpful. The latter assessment should evaluate, besides bacterial morphotypes, their quantity, heterogeneity, inflammatory infiltrate, and quantification of parabasal cells.

## Author Contributions

JE and AG conceived and designed the study. NO, AL, JB, and AS, drafted and revised the article where appropriate. NO, AL, and JB prepared the figures. AG and JE carried out the final revision of the manuscript. All authors contributed to the article and approved the submitted version.

## Conflict of Interest

The authors declare that the research was conducted in the absence of any commercial or financial relationships that could be construed as a potential conflict of interest.

## Publisher's Note

All claims expressed in this article are solely those of the authors and do not necessarily represent those of their affiliated organizations, or those of the publisher, the editors and the reviewers. Any product that may be evaluated in this article, or claim that may be made by its manufacturer, is not guaranteed or endorsed by the publisher.
